# Dental Implants with Anti-Biofilm Properties: A Pilot Study for Developing a New Sericin-Based Coating

**DOI:** 10.3390/ma12152429

**Published:** 2019-07-30

**Authors:** Paolo Ghensi, Elia Bettio, Devid Maniglio, Emiliana Bonomi, Federico Piccoli, Silvia Gross, Patrizio Caciagli, Nicola Segata, Giandomenico Nollo, Francesco Tessarolo

**Affiliations:** 1 Department of Cellular, Computational and Integrative Biology, University of Trento, 38122 Trento, Italy; 2 Department of Industrial Engineering, University of Trento, 38122 Trento, Italy; 3 Department of Laboratory Medicine, Azienda Provinciale per i Servizi Sanitari, 38122 Trento, Italy; 4 Department of Chemical Sciences, University of Padova, 35131 Padova, Italy; 5 Healthcare Research and Innovation Program (IRCS-FBK-PAT), Bruno Kessler Foundation, 38122 Trento, Italy

**Keywords:** dental implants, biofilm, coating, titanium (Ti), Ti-6Al-4V, bioconjugation

## Abstract

Aim: several strategies have been tested in recent years to prevent bacterial colonization of dental implants. Sericin, one of the two main silk proteins, possesses relevant biological activities and also literature reports about its potential antibacterial properties, but results are discordant and not yet definitive. The aim of this study was to evaluate the effectiveness of different experimental protocols in order to obtain a sericin-based coating on medical grade titanium (Ti) able to reduce microbial adhesion to the dental implant surface. Materials and Methods: different strategies for covalent bonding of sericin to Ti were pursued throughout a multi-step procedure on Ti-6Al-4V disks. The surface of grade 5 Ti was initially immersed in NaOH solution to obtain the exposure of functional -OH groups. Two different silanization strategies were then tested using aminopropyltriethoxysilane (APTES). Eventually, the bonding between silanized Ti-6Al-4V and sericin was obtained with two different crosslinking processes: glutaraldehyde (GLU) or carbodiimide/N-Hydroxy-succinimide (EDC/NHS). Micro-morphological and compositional analyses were performed on the samples at each intermediate step to assess the most effective coating strategy able to optimize the silanization and bioconjugation processes. Microbiological tests on the coated Ti-6Al-4V disks were conducted in vitro using a standard biofilm producer strain of *Staphylococcus aureus* (ATCC 6538) to quantify the inhibition of microbial biofilm formation (anti-biofilm efficacy) at 24 hours. Results: both silanization techniques resulted in a significant increase of silicon (Si) on the Ti-6Al-4V surfaces etched with NaOH. Differences were found between GLU and EDC/NHS bioconjugation strategies in terms of composition, surface micro-morphology and anti-biofilm efficacy. Ti-6Al-4V samples coated with GLU-bound sericin after silanization obtained via vapor phase deposition proved that this technique is the most convenient and effective coating strategy, resulting in a bacterial inhibition of about 53% in respect to the uncoated Ti-6Al-4V disks. Conclusions: The coating with glutaraldehyde-bound sericin after silanization in the vapor phase showed promising bacterial inhibition values with a significant reduction of *S. aureus* biofilm. Further studies including higher number of replicates and more peri-implant-relevant microorganisms are needed to evaluate the applicability of this experimental protocol to dental implants.

## 1. Introduction

Bacterial biofilm formation on oral surfaces constitutes the starting point of the most common oral diseases, including caries, gingivitis, periodontitis and, when dental implants are involved, mucositis and peri-implantitis [1,2]. Peri-implantitis is considered today a major problem in dentistry and it is of growing importance as the number of patients treated with implant therapy increases. In a recent systematic review, Derks and Tomasi reported a prevalence of peri-implantitis of 22% (95% confidence interval, 14% to 30%) [3]. Untreated peri-implantitis can lead to detrimental effects on osseointegration, ultimately resulting in the so called “biological” implant failure (in opposition to “mechanical” failure), since it is related to biological processes [4,5,6,7,8,9]. The treatment of this pathology is arduous and constitutes an important issue in modern clinical practice. Prevention strategies have therefore become extremely important. Numerous scientific studies are nowadays focused on the development of new methods to prevent bacterial adhesion and implant infection [10].

Numerous techniques have been developed over the years to coat or chemically modify Ti implantable surfaces for dental applications in order to improve the implant response to oral microbiota. The objective of these interventions, in most cases, is achieved as a secondary effect for having enhanced the osseointegration process [11,12,13,14,15,16,17,18,19]. The risk of an early implant infection is indeed reduced by shortening the time for eukaryotic cells adhesion and osseointegration. This approach has been revealed to be effective for protecting the implant immediately after implantation, but has a comparably lower impact on microbial colonization and infections occurring late after implant placement.

The coating of dental implants with bioactive molecules is a recent approach to modify pristine Ti biochemical properties and hinder bacterial colonization of implant surface [20]. Implant coatings are essentially based on two strategies: drugs or biomolecules are mixed within the bulk of the device and released through diffusion or degradation of the matrix, or they can be grafted to the implant surface [21]. With the purpose of preventing implant infection, these strategies have been implemented using different drugs and molecules or constructs to obtain anti-bio-adhesive coatings, antimicrobial coatings (e.g., Vancomycin, Ag, Zn) or coatings (e.g., calcium phosphate, polylactic acid, chitosan) with controlled release of antimicrobial agents [22]. 

A natural biomolecule which has been shown to possess interesting biological characteristics is silk sericin. Silk produced by the silkworm is composed of two main proteins: the fibroin which forms the internal part and has a structural function, and sericin, a family of rubber-like coating proteins which constitutes 25–30% of the weight of the silk secreted by the *Bombyx mori* worm [23].

Sericin is currently a waste product of the textile silk industry since it is removed from fibroin in a process called “degumming”, to allow fiber acquiring luster, softness, smoothness, whiteness and dyeability. Sericin has been recently revalued due to its properties when in contact with biological materials, making it usable in the pharmacological, cosmetic and biotechnological fields. While some positive biological effects, as for example antioxidant or anti-inflammatory behavior, have been proven, the anti-bacterial efficacy of sericin is still debated and it is described by studies that often provide contrasting results [24,25,26]. 

Nuchadomrong et al. evaluated the influence of different degumming methods on the anti-bacterial capacity of the *Samia ricini* sericin comparing it with the *Bombyx mori* sericin. *Escherichia coli, Salmonella choleraesuis and Bacillus subtilis* strains were found to be susceptible, at certain concentrations, to both sericins, while only the *S. ricini* sericin showed an inhibitory effect on *S. aureus* [27]. Other studies reported an anti-bacterial effect of *S. ricini* [28] and *B. mori* sericin [29,30,31].

Nevertheless, Seves et al. observed that the raw silk buried in the ground has a bacterial growth greater than the degummed one, supposing that this result was due to the presence of sericin that is used by the bacteria for their growth [32]. Similarly, Akiyama et al. they found the growth of gram-positive bacteria on the silk thread used for stitching mouse skin [33]. Kaur et al. conducted in vitro studies using gram-negative *E. coli* bacteria in order to determine if some components of the cocoon resist the colonization of microorganisms. Their results demonstrate that no component of the cocoon (including sericin) hinders the growth of such bacteria suggesting that the previously reported anti-bacterial properties are actually derived from chemicals used to separate or purify the elements of the cocoon [34].

The aim of this study was to evaluate in vitro the effectiveness of different experimental protocols to obtain a sericin-based coating on medical grade Ti able to reduce microbial adhesion and biofilm formation on the dental implant surface.

## 2. Materials and Methods

### 2.1. Study Design

**Figure 1:**
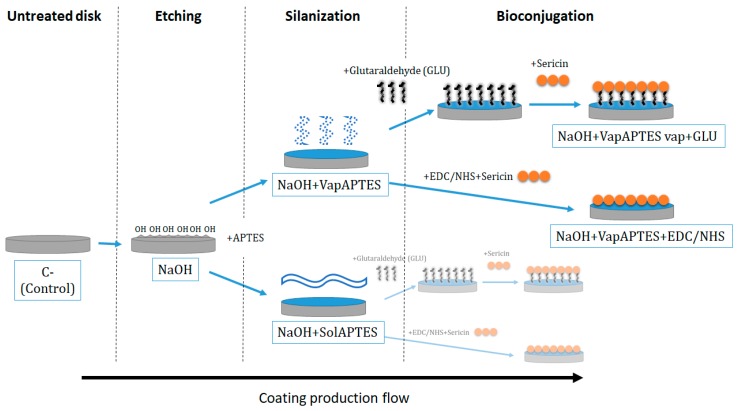
Diagram of the four investigated coating strategies obtained by combining different silanization (vapor-phase deposition, VapAPTES or organic/aqueous solvent, SolAPTES) and bio conjugations (glutaraldehyde (GLU) or carbodiimide/N-Hydroxy-succinimide (EDC/NHS)) process after a preliminary etching phase (NaOH).

This *in-vitro* study was composed by two main phases. In the first phase, a multi-step procedure was implemented with the aim to bond covalently the sericin to the Ti-6Al-4V disk surface. In this procedure, after a preliminary alkaline etching conditioning of the Ti-6Al-4V surface, four different coating strategies were implemented combining two different silanization techniques and two different bioconjugation processes (Figure 1). The coupling agent was a silane (APTES), covalently bonded to the hydroxyl groups of the Ti-6Al-4V oxidized surface, previously exposed via chemical etching. Eventually sericin was immobilized to the silanes functional groups by using either a cross-linker (glutaraldehyde) or catalyzing a direct covalent bonding with EDC/NHS. We followed and checked morphological and compositional evolution of the Ti-6Al-4V surface at every step by realizing a battery of instrumental characterization techniques such as SEM, EDXS, AFM and XPS.

With the aim of controlling the process in an incremental way, samples were produced and analyzed after both an intermediate step of preparation and at completion of the coating strategies. Based on the results obtained from micromorphological and compositional analysis, the best performing coating strategy has been identified. Disks produced according to the best protocol were further checked for their ability to inhibit formation of microbial biofilm at their surface in a controlled microbiological experiment with a standard strain recognized for its ability to form biofilm. 

### 2.2. Chemicals

Sericin (Pure Sericin^TM^ Seiren Co., Ltd., Tokyo, Japan), aminopropyltriethoxysilane, carbodiimide, N-Hydroxy-succinimide, and glutaraldehyde (Sigma Aldrich, St Louise, MO, USA) were analytical grade. 

### 2.3. Ti-6Al-4V Substrate Preparation 

Ti-6Al-4V alloy grade 5 disks (CLC scientific, Vicenza, Italy) 10 mm in diameter and 2 mm in thickness were produced by computer numerical control machining and cleaned using the same standards for dental abutments.

The disks were polished with increasing fine-grained abrasive paper up to 4000 grit to remove any possible manufacturing irregularities and undertook three washing steps to remove any impurity and grinding residues. Washing was realized by sonication for 15 min each in three consecutive solutions: acetone, 70% ethanol in double distilled water (ddw) and pure ddw. 

### 2.4. NaOH Etching

The disks were immersed for 24 h in a 2.5 M solution of sodium hydroxide (NaOH) at 60 °C to expose −OH functional groups. NaOH etched disks were then thoroughly washed twice in ddw.

### 2.5. Silanization

Silanization with aminopropyltriethoxysilane (APTES) was realized according to two different protocols: i) deposition in organic/aqueous solvent (SolAPTES); and ii) vapor-phase deposition (VapAPTES).

SolAPTES deposition was realized by immersing the etched disks in a solution of water (5% v/v) and APTES (5% v/v) in ethanol at pH 4.5–5.5 for 2 h. The silanized samples were then sonicated in ethanol to remove the excess of physio-absorbed molecules and cured at 110° in air for 30 min. VapAPTES was realized by exposing etched disks to a saturated vapor of APTES for 24 h at room temperature.

### 2.6. Bioconjugation

The covalent bond between silane and sericin was obtained by either using glutaraldehyde (GLU) or carbodiimide/N-Hydroxy-succinimide (EDC/NHS). 

The GLU bioconjugation strategy was obtained by immersing silanized disks in a solution of glutaraldehyde (2% v/v in ddw) for 24 h at room temperature. The disks were then washed in ddw and transferred into a solution of sericin (1% w/v in ddw) at 37 ° C for 2 h.

Bioconjugation using EDC/NHS was realized by immersing silanized disks in a sericin solution (10 mg/mL) in PBS at pH 7.4. EDC and NHS were then added into the solution up to a final concentration of 0.1 M and 0.025M respectively and left for 2 h at room temperature.

Sericin coated samples were rinsed twice in ddw to remove the unattached sericin protein.

### 2.7. Micromorphological and Compositional Surface Characterization

Micromorphology and composition of disks at each intermediate production step and after completion of all different sericin coating strategies reported in Figure 1 were studied by multiple investigation techniques. A XL30 ESEM FEG (FEI-Philips, Eindhoven, The Nederland) scanning electron microscope (SEM) equipped with energy dispersive X-rays spectroscopy (EDXS) (EDAX, Mahwah, NJ, USA) was used to investigate microstructure and elemental composition of the samples surface. Secondary electrons and backscattered electrons images were collected at magnifications ranging from 50 to 20,000 times using low energy primary electron beam. Surface topography was measured by Atomic Force Microscopy (AFM, Solver pro, NT-MDT Spectrum Instruments Moscow, Moscow, Russia) using a silicon nitride tip (NT-MDT NSG-11B, 10 nm tip radius, 181 kHz resonance frequency), scanning areas 20 µm × 20 µm wide in semi-contact mode. X-ray Photoelectron Spectroscopy (XPS) was also used to have a more quantitative evaluation of the elemental composition at the sample surface.

Samples were investigated by XPS measurements with a Φ 5600ci Perkin–Elmer spectrometer, using a standard aluminum (Al Kα) source, with an energy of 1486.6 eV operating at 200 W. The X-ray source employed was located at 54.7° relative to the analyzer axis. The working pressure was < 5 × 10^–8^ Pa. The calibration was based on the binding energy (B.E.) of the Au4f7/2 line at 83.9 eV with respect to the Fermi level. The standard deviation for the B.E. values was 0.15 eV. The reported B.E. were corrected for the B.E. charging effects, assigning the B.E. value of 284.6 eV to the C1s line of carbon [35]. Survey scans were obtained in the 0–1350 eV range (pass energy 58.7 eV, 0.5 eV/step, 25 ms/step). Detailed scans (11.75–29.35 eV pass energy, 0.1 eV/step, 50–150 ms/step) were recorded for relevant regions (O1s, C1s, Si2p, N1s, Na1s, Ti2p) depending on the sample. The atomic composition, after a Shirley-type background subtraction, [36] was evaluated using sensitivity factors supplied by Perkin–Elmer [37]. Assignment of the peaks was carried out according to data from the literature and available database [38].

Comparative data analysis was performed along the production flow to reveal the added value of each incremental step (e.g., C− VS NaOH) and across different process options to identify the most effective treatment (e.g., NaOH + SolAPTES VS NaOH + VapAPTES).

### 2.8. Microbiological Test

A microbiological test was designed and performed using a bioreactor model to stimulate the sessile adhesion and growth of a biofilm former microbial strain. In order to quantify the surface ability to inhibit the biofilm adhesion and formation (anti-biofilm activity), microbiological tests were conducted in parallel on untreated Ti-6Al-4V disk and on disk subjected to intermediate and complete treatment for sericin coating. Namely, the following samples were produced for microbiological testing: untreated controls (C−), NaOH etched disks (NaOH), etched and vapor phase silanized disks (NaOH + VapAPTES), etched silanized and sericin coated disk using glutaraldehyde (NaOH + VapAPTES + GLU), and etched silanized and sericin coated disk using carbodiimide/N-Hydroxysuccinimide (NaOH + VapAPTES + EDC/NHS). 

Tests were conducted in triplicate using 24-well polystyrene plates. *Staphylococcus aureus* ATCC 3568 was used as a recognized standards strain able to form thick biofilm. A refrigerated strain stock was thawed and plated on sheep blood agar plate. Colonies obtained after incubation at 36 °C for 24 h have been used to prepare a bacterial suspension with a final concentration of 10^7^ CFU/mL in trypticase soy broth supplemented with 1% dextrose. One disk per well was positioned using sterile pliers. An aliquot of 1 mL of bacterial suspension was poured over each disk. The plate was covered with lid and incubated for 24 h at 37 °C. At the end of the incubation period, the bacterial suspension was removed using a pipette and the disks were gently washed three times adding 1 mL of PBS per each well in order to remove any planktonic or poorly adherent microbial cell from the disk surface. To quantify the adherent biofilm biomass, each disk was then immersed into 1 mL of crystal violet (0.2% in water) for 10 min. Disks were then removed from the dye solution, immersed twice in water to remove excess of dye and allowed to dry for about 1 h under a fume hood. Dried disks were moved in a new multi-well plate and 1 mL of acetic acid solution (33% in water) was added to each well in order to resuspend the biofilm-related dye. Dye concentration, representative of the biofilm biomass, was quantified spectrophotometrically by quantifying absorbance at 570 nm.

Absorbance data were averaged over triplicate samples and presented as mean and standard error. A relative amount of biofilm biomass was presented as percentages by normalizing data to the absorbance value of the untreated controls. Inhibition efficacy was calculated by using the following formula.

*Inhibition* (%) = 100 − *BiofilmBiomass* (%) (1)

Absorbance data obtained from NaOH + VapAPTES + GLU and NaOH + VapAPTES + EDC/NHS disks was compared to untreated control for statistical significance. Student-t test was used with a significance for *p* < 0.05.

## 3. Results

### 3.1. Morphology and Surface Composition

#### NaOH Etching

**Figure 2:**
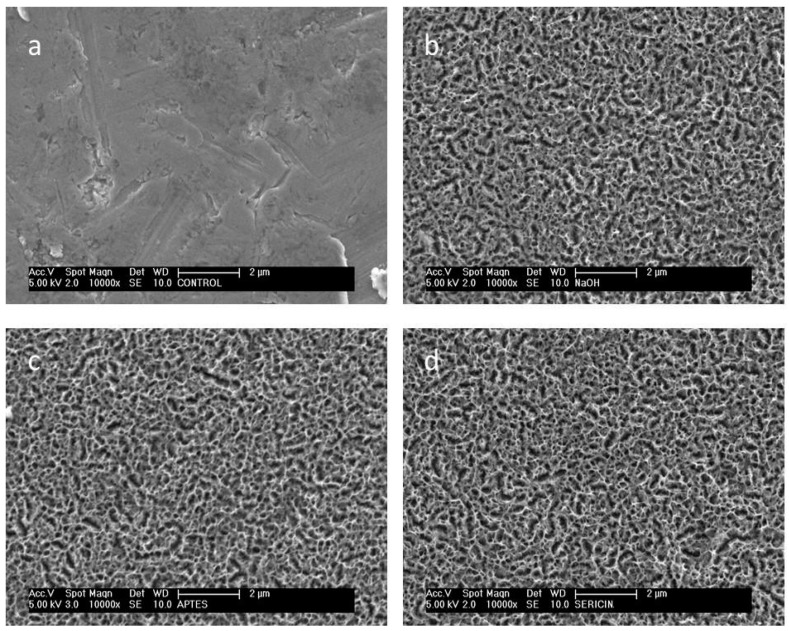
Micromorphology of the disk surface at different production step: (**a**) untreated control, (**b**) after etching (NaOH), (**c**) after etching and silanization (NaOH + VapAPTES), (**d**) after etching, silanization and sericin bioconjugation with glutaraldehyde (NaOH + VapAPTES + GLU). Images (**c**,**d**) are representative also for the micromorphology of disks subjected to the alternative silanization (SolAPTES) and bioconjugation (EDC/NHS) processes respectively. Scanning electron microscopy, secondary electrons detector signal, original magnification 10,000×.

**Figure 3:**
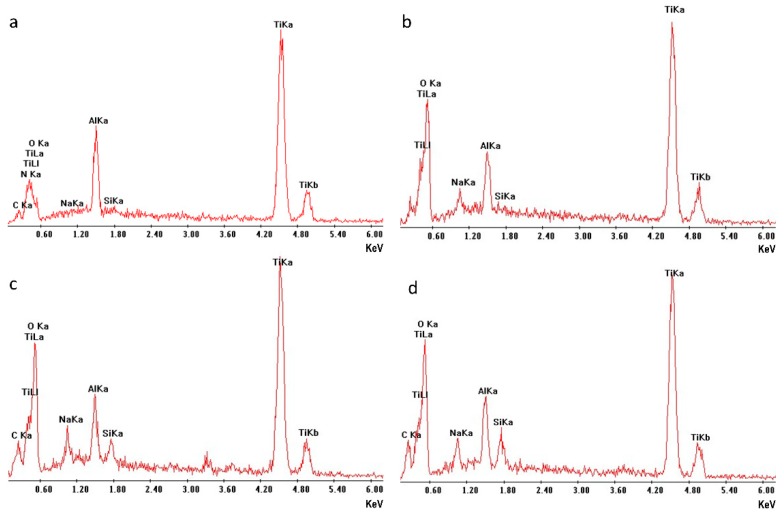
Surface compositional analysis of Ti-6Al-4V disks subjected to different surface treatments: (**a**) untreated control, (**b**) after etching (NaOH), (**c**) after etching and silanization by vapour deposition (NaOH + VapAPTES), (**d**) after etching and silanization by solvent deposition (NaOH + SolAPTES). Sodium (Na) and oxygen (O) are introduced by the etching phase and Silicon (Si) by silanization phase. Energy dispersive spectroscopy. Primary electron beam energy was set to 15 KeV. Spectra are collected over an area of 25 µm^2^. Integration time 200 s.

The untreated sample (Figure 2a), presented a uniform surface to the SEM analysis with minor parallel grooves related to the polishing with carbide sandpaper. Surface composition assessed by EDX (Figure 3a) is typical for an environmentally oxidized Ti6Al4V alloy.

**Figure 4:**
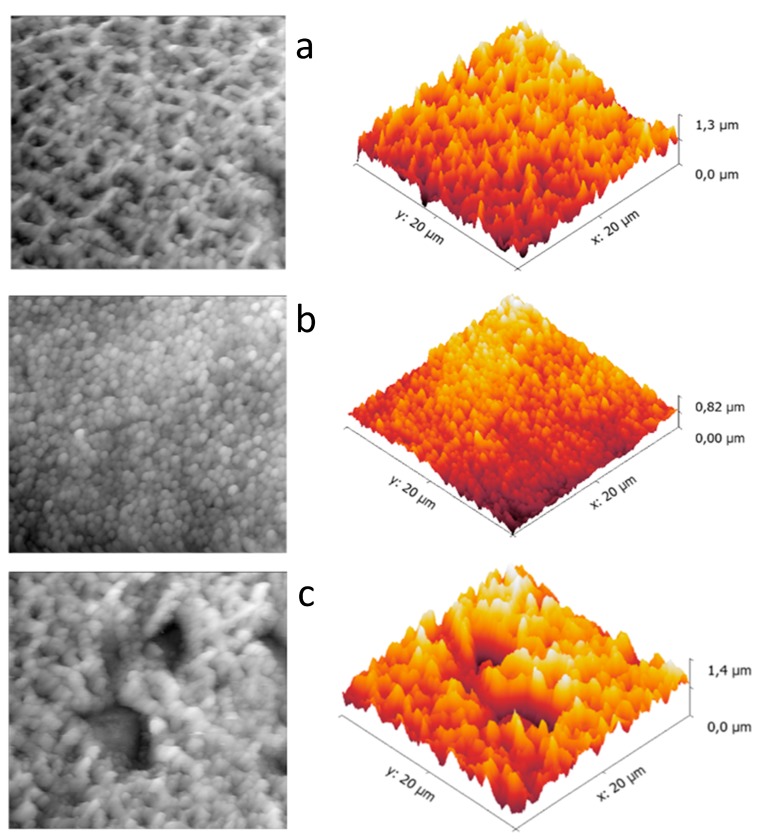
Nano-morphology (2D images on the left and 3D reconstructions on the right) of the disk surface at different production step: (**a**) after etching and silanization (NaOH + VapAPTES), (**b**) after etching, silanization and sericin bioconjugation with glutaraldehyde (NaOH + VapAPTES + GLU), (**c**) after etching, silanization and sericin bioconjugation with EDC/NHS (NaOH + VapAPTES + NDC/NHS). Atomic force microscopy, semi-contact mode.

The NaOH treatment induced a clear morphological change at the surface, showing the creation of micro and nano-crevices related to the chemical etching of the NaOH solution. The new micro and nano-morphology was diffused and uniform over the whole disk surface. Representative images were obtained by SEM (Figure 2b) and AFM (Figure 4a), respectively. Comparison of compositional analysis before and after NaOH treatment, evidenced a substantial increase in surface oxidation and the appearance of sodium among the minor surface components. Qualitative EDX spectra are reported in Figure 3a,b for the untreated and NaOH etched surfaces, evidencing a higher peak for the oxygen and the appearance of a sodium peak in the NaOH sample. Compositional atomic percentages obtained with XPS on NaOH etched surface were 52.5% and 11.7% for O and Na respectively (Table 1), showing a relevant increase, as expected, for sodium and a corresponding increase also of Ti (from 5 to 17.6%), this latter related to the etching of the surface contamination (C% from 58.2% to 16.9%). 

### 3.2. Silanization

**Figure 5:**
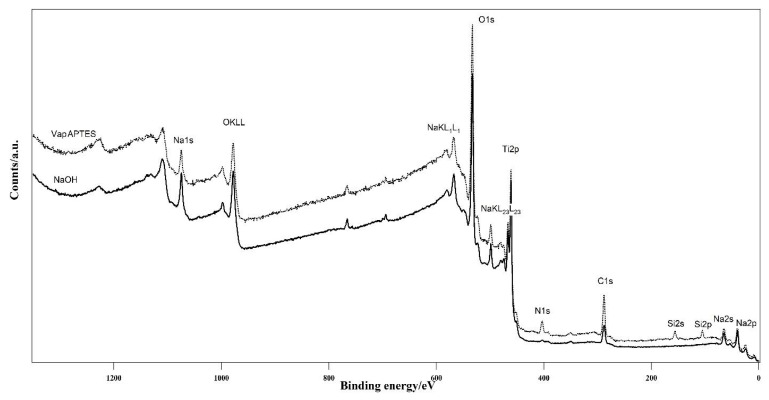
Comparison of the survey spectra of the samples after NaOH etching but before functionalization (NaOH) and after aminopropyltriethoxysilane (APTES) (VapAPTES) functionalization. Successful attachment of APTES is evidenced by the presence of nitrogen and silicon species, which were not present on the bare Ti-6Al-4V surface.

Both VapAPTES and SolAPTES silanization processes did not resulted in further changes of the surface morphology as shown in Figure 2c and Figure 4a. Regardless, a clear increase of silicon was documented. Qualitative spectra obtained at EDX showed the presence of the Si peak. Figure 3c,d reports qualitative compositional data for disks treated according to NaOH + VapAPTES and NaOH + SolAPTES protocols, respectively. Quantitative compositional analysis performed with XPS reported an atomic percentage of Si of 4.4% and 2.9% for samples underwent to silanization by vapor or solvent deposition (Table 1), respectively, therefore showing a more efficient silanization by vapor phase. By XPS, an increase in nitrogen and carbon, both elements present in the APTES molecule, was also detected, therefore demonstrating the anchoring of the APTES molecule. In Figure 5, the surveys corresponding to NaOH + VapAPTES and NaOH + SolAPTES samples are exemplarily superimposed. Based on the compositional data and considering that VapAPTES protocol resulted in a lower risk of surface contamination and required a lower volume of APTES, silanization via vapor phase deposition was considered for the following bioconjugation procedures in this study. 

### 3.3. Bioconjugation

The two bioconjugation strategies implemented in the study resulted in minor or non-detectable micro-morphological differences at SEM (Figure 2d). However, appreciable nano-morphological changes were visible at AFM images (Figure 4b,c). The sharp peaks induced by NaOH treatment were still present after the silanization process (Figure 4a), but appeared smoothed and rounded after sericin bioconjugation, in agreement with the expected deposition of the protein on a globular form. The low electron density of the protein layer did not permit proper SEM imaging. On the contrary, AFM images obtained in semi-contact mode, allowed reliable imaging of soft surface organic structures. Moreover, AFM elicited a uniform morphological pattern on the NaOH + VapAPTES + GLU samples (Figure 4b), while evidencing areas with different morphologies at the surface of the NaOH + VapAPTES + EDC/NHS samples (Figure 4c). Results of the compositional analysis realized by XPS on the bioconiugated surfaces are supportive of sericin deposition (Table 1). The protein bioconjugation resulted in different extent by using GLU or NHS/EDC protocols. NaOH + VapAPTES + GLU samples showed a clear reduction in the atomic percentages for Ti and silicon, and an increase of the amount of nitrogen and carbon (Table 1), suggesting the effective coverage of the Ti-6Al-4V surface with the anchored molecules. Being XPS a surface sensitive method, the contribution of only the outmost layers is enhanced. On the other hand, compositional values for NaOH + VapAPTES + EDC/NHS samples evidenced no major variations in the same elements, in this case instead suggesting that the Ti-6Al-4V surface was not effectively derivatized, this is outlined also by the relatively low silicon content. In summary, XPS results were in agreement with the AFM findings, showing a more effective and uniform protein coating after GLU bioconjugation protocol, but uneven coating after EDC/NHS protocol. 

### 3.4. Anti-Biofilm Activity

The bioreactor model was able to promote the *Staphylococcus aureus* biofilm formation at the disks surface within 24 h of incubation. Absorbance values at 570 nm varied among the different tested samples in the range between 1.11 and 2.38. Mean absorbance values and standard errors over the three replicates are reported in Table 2. 

The highest absorbance value was obtained for the untreated disks, confirming the absence of any antibacterial and antibiofilm property of controls. The biofilm biomass percentage of controls was then set to 100% and inhibition values were calculated with respect to controls (Table 2).

**Figure 6:**
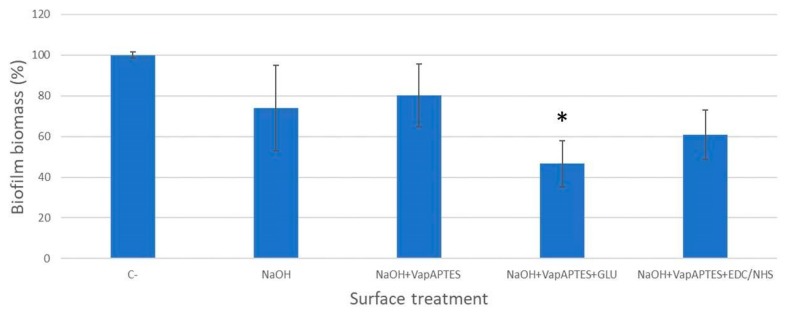
Biofilm biomass (%) of *Staphylococcus aureus* ATCC 6538 formed at 24 h of incubation on the surface of Ti-6Al-4V disks subjected to different surface treatments. Data are normalized to untreated controls (C−). *p < 0.05.

Values of the biofilm biomass obtained for all tested samples are reported in Figure 6, for ease of comparison. Although minor variation in the absorbance and biofilm biomass could be related to the NaOH etching and to the silanization process, the lowest values were obtained from the samples subjected to sericin bioconjugation. 

Statistical comparison between untreated samples and bioconjugated disks evidenced a statistically significant difference (*p* = 0.041) in the absorbance values of the NaOH + VapAPTES + GLU treated disks, documenting a biofilm inhibition activity of 53% for the process based on the glutaraldehyde bioconjugation of sericin. 

## 4. Discussion

The present work was aimed at coupling a functional biomolecule to the Ti-6Al-4V functionalized surface. Although part of the available dental implants are manufactured using commercially pure Ti, Ti4Al6V was chosen as representative material in this pilot study considering this alloy is often used for both dental implant and transmucosal components (e.g., implant abutments), with these involved in preserving the mucosal seal from bacterial infiltration.

In literature the activation of the surface with NaOH is reported to have a double function: allowing to incorporate −OH groups, thus making the surface more hydrophilic, and increasing the micro- and nano-surface roughness [39,40,41,42]. An increase in surface energy and consequently an increased bioactivity [43] has also been reported. In the present work, we highlighted a micro and nano-morphological modification by SEM and a compositional enrichment in oxygen by XPS and EDXS that is consistent with the expected increase of hydroxyl groups. The presence of adventitious carbon at this stage was considered a natural contaminant in the XPS analysis and is commonly used for the calibration of the instrument [44]. By contrast, sodium was an unexpected element found by both EDX and XPS on the Ti-6Al-4V surface after the NaOH etching. Although the sodium concentration could be probably reduced by extending or improving the washing procedure after the NaOH treatment, remnants did not appear to have interfered with the silanization reactions and bioconjugation.

Silanization is the most used and consolidated method for immobilizing biological molecules on model inorganic surfaces [45]. In this study, APTES was linked to the activated Ti-6Al-4V surfaces using two strategies: deposition in organic-aqueous solvent and vapor deposition. The effectiveness of both techniques was documented by the increase of silicon using both XPS (Table 1) and EDXS (Figure 3). It should be outlined that, whereas XPS is surface sensitive and its sampling depth is limited to the first few nanometers, EXDS instead probes a sampling depth which is much higher, reaching the micrometer range. This treatment did not introduce substantial micro-morphological modification according to the SEM and AFM investigations. The microstructure induced by the NaOH etching was still present after both the silanization tested strategies.

The mere physical adsorption of proteins at the Ti surface is not suitable for use in the oral environment because fluids that are present at the peri-implant region can easily remove the protein by spreading it away from the implant site, especially in the case of a water-soluble protein such as sericin. Furthermore, it has been found that physical adsorption can lead to a conformational change of the protein denaturing it and altering its properties [46,47,48,49,50,51]. On the other end, the covalent bond represents a valuable option for a stable and long-lasting attachment, with promising properties towards early and late implant microbial complications. 

In order to covalently immobilize biological material on substrates bearing functional groups, coupling with terminal amino groups has been frequently used. The use of glutaraldehyde as a coupling agent provides satisfactory results for immunocytochemistry or in situ hybridization and for the maintenance of biological activity of a given molecule in vitro [50,51,52,53]. Other studies have shown that the combined use of silanization and glutaraldehyde are effective techniques to immobilize immunoglobulins on glass [54]. In this study, the use of glutaraldehyde appeared to be effective in binding the protein to the silane. The increase in carbon and the decrease in Ti at XPS after the bioconjugation with glutaraldehyde confirm this hypothesis and the AFM analysis shows uniformly distributed globular aggregates on the surface.

The other bioconjugation technique used here was based on crosslinking via carbodiimides. The reaction mediated by EDC converts the carboxyl group into an intermediate of O-acylisourea which readily reacts with the amino group, resulting in covalent amide bond between the biomolecules and the surface [55]. The effectiveness of protein conjugation was investigated by XPS analysis and showed the achievement of a less effective protein coating with a lower masking action of the underlying Ti-6Al-4V substrate. This was further confirmed by AFM recognition of an irregularly patterned surface, including areas without the sericin aggregate at the surface. The AFM analysis also shows jagged peaks and large depressions that make the surface much less uniform than the bioconjugate samples with glutaraldehyde by suggesting the exposure of the Ti-6Al-4V surface in some areas. In summary, these results evidenced that Ti-6Al-4V samples in this study were more effectively coated by sericin using the NaOH + VapAPTES + GLU protocol and by using the vapor-based approach. Microbiological test provide further evidence of the higher effectiveness of this coating protocol for obtaining an antibiofilm surface.

Considering the contrasting results reported in the literature about antibacterial properties of sericin and that characteristics other than antibacterial effect could be responsible for an antibiofilm property, it was decided to design and carry out a specific test to explore the anti-biofilm properties of the pure sericin coating. The realized biofilm model (adapted from Ceresa et al. [56]) allowed the development of *S. aureus* (ATCC 6538) microbial biofilms at the surface of Ti-6Al-4V disks and allowed the differences between sericin-coated and untreated surfaces to be evidenced. The choice to use *S. aureus* as a model microorganism was due its proven ability in forming a thick and reproducible biofilm at the Ti surface in laboratory setting. 

Ti-6Al-4V disks coated with glutaraldehyde-bound sericin resulted in a significant biofilm inhibition of approximately 53% compared to the untreated Ti-6Al-4V. Samples obtained by bioconjugating sericin via EDC/NHS showed a lower inhibition possibly due to the inhomogeneous and incomplete surface coating.

The exact anti-biofilm inhibition mechanism of the sericin-based coating on *S. aureus* biofilms is not yet completely known but it seems to be related to the damage of the cell membrane. After exposure to sericin, in fact, the integrity of the membrane is weakened and the metabolism is blocked, thereby eventually inhibiting the growth and reproduction of *S. aureus.*

Further experiments must be carried out in order to support the data so far produced and to better understand the most critical points of the coating protocol that can reduce the anti-biofilm effectiveness.

## 5. Conclusions

Both aqueous-organic solvent and vapor phase treatments were found effective for the surface silanization. Information provided by compositional and morphological analysis suggest that the use of glutaraldehyde can be more effective in forming a uniform protein coating on Ti-6Al-4V, while the use of EDC-NHS did not produce satisfactory results in terms of uniform protein coating.

The coating with glutaraldehyde-bound sericin after silanization in the vapor phase showed promising bacterial inhibition values with a significant reduction of *S. aureus* biofilm. Further studies including a higher number of replicates and a range of peri-implant relevant microorganisms are needed to evaluate the applicability and exploitability of this experimental protocol during dental implants production. 

## Figures and Tables

**Table 1:** Surface atomic percentages on samples at different steps of the production process. Composition was obtained for the most relevant elements identified by XPS.

Surface Treatment	C 1s	N 1s	O 1s	Na 1s	Si 2p	S 2p	Ti 2p
C-	58.2		34.1	1.5		1.15	5.0
NaOH	16.9	0.9	52.5	11.7	0.4		17.6
NaOH + VapAPTES	26.1	5.4	49.8		4.4		14.4
NaOH + SolAPTES	22.5	3.5	55.0		2.9		16.1
NaOH + VapAPTES vap + GLU	49.6	8.5	33.8		2.4		5.7
NaOH + VapAPTES vap + EDC/NHS	22.0	5.3	54.7		0.5		17.5

**Table 2:** Results summary of the microbiological tests. Mean and standard error (S.E.) values of the biofilm absorbance measured in triplicate experiments at different steps of the production process. Estimated biofilm biomass and inhibition percentages are calculated in respect to the amount of biofilm grown on controls.

Surface Treatment	Absorbance @540 nm		Biofilm Biomass (% in Respect to Controls)	Biofilm Inhibition (% in Respect to Controls)
	Mean	S.E.	Mean	Mean
C-	2.38	0.04	100	0
NaOH	1.76	0.50	74	26
NaOH + VapAPTES	1.91	0.37	80	20
NaOH + VapAPTES vap + GLU	1.11	0.27	47	53
NaOH + VapAPTES vap + EDC/NHS	1.45	0.29	61	39
